# Complete mitochondrial genome of golden variant of freshwater fish *Labeo rajasthanicus* (Cypriniformes: Cyprinidae): endemic to India

**DOI:** 10.1080/23802359.2023.2290128

**Published:** 2023-12-12

**Authors:** Mamta Singh, Ved Prakash Saini, Vindhya Mohindra, Manohar Lal Ojha, Kuldeep Kumar Lal, Rajeev Kumar Singh

**Affiliations:** a College of Fisheries, Bihar Animal Sciences University, Kishanganj, India; b ICAR-National Bureau of Fish Genetic Resources, Lucknow, India; c College of Fisheries, Maharana Pratap University of Agriculture & Technology, Udaipur, India; d ICAR-Central Institute of Brackishwater Aquaculture, Chennai, India

**Keywords:** *Labeo rajasthanicus*, mitochondrial genome, phylogenetics

## Abstract

The complete mitochondrial genome of the freshwater fish species *Labeo rajasthanicus* was obtained, using Illumina NovaSeq 6000 with 2 × 150 bp paired-end sequencing. The mitogenome of *L. rajasthanicus* is 16,738 bp in length (GenBank accession no.: OQ834146), comprised of 13 protein-coding genes, 22 tRNA genes, two rRNA genes, and a control region, i.e. D-loop. The arrangement of genes was found to be identical to other Cypriniformes fish mitogenome, available in the NCBI database. The taxonomic status of *L. rajasthanicus* as a valid species was debated by some researchers and it was considered a synonym of *L. boggut.* However, phylogenetic analysis in the present study supports the species validity of *L. rajasthanicus*, as it showed a distinct node well separated from *L. boggut* and supported by a high bootstrap value. Furtherly, the pairwise genetic divergence among studied species showed the divergence between *L. rajasthanicus* and *L. boggut* as 1.6% whereas the minimum divergence was found to be 0.13% with *L. dussumieri* followed by *L. fimbriatus* (0.58%) and *L. gonius* (0.63%). The complete mitogenome of *L. rajasthanicus* will also be useful as a baseline reference genome for the reconstruction and annotation of the mitogenome of other *Labeo* species.

## Introduction

*Labeo rajasthanicus* (Datta and Majumdar 1970) belongs to the order Cypriniformes, family Cyprinidae, and subfamily Labeoninae, and is a medium-sized carp endemic to the Indian state of Rajasthan. It was first described from Jaisamand Lake of Rajasthan based on a single specimen in 1970. The species validity of *L. rajasthanicus* was debated as Jayaram and Dhas (2000) considered this species a synonym of *Labeo boggut* due to its phenotypic similarity. After more than four decades of confusion about its validity as a species, *L. rajasthanicus* was confirmed as a valid species in the year 2015 with the designation of a neotype (Lal et al. 2015). This species was evaluated as an important minor carp with great potential for inclusion in composite fish culture (Bansal et al. 2014). Due to its restricted distribution, this species has conservation value, and its golden variant holds great ornamental value. A farm-type golden variant of *L. rajasthanicus* (Pratap Sunahari) was also developed through captive breeding, which was recommended to be promoted as an ornamental fish, especially for garden pools (Saini et al. 2022). However, its inclusion in the culture system, conservation in native habitats, and ornamental trade require a thorough evaluation of its genetic distinctiveness and diversity. Mitochondrial gene sequences are widely used to determine species diversity and evolutionary divergence among species. Therefore, the present study was conducted to generate the complete mitogenome sequence of *L. rajasthanicus* to decipher its structural characteristics and establish the phylogenetic relationship with closely related species.

## Materials and methods

**Figure 1.**
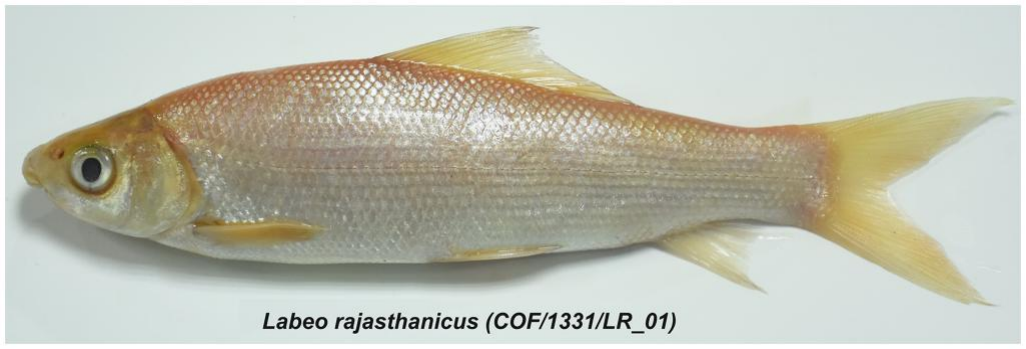
Image of voucher specimen of *Labeo rajasthanicus* with specimen repository accession number COF/1331/LR_01 (source of image: original image taken by author Mamta Singh).

The live specimen of the golden variant of *L. rajasthanicus* was obtained from the culture pond of the College of Fisheries, Maharana Pratap University of Agriculture & Technology, Udaipur, Rajasthan, India (24.5788 N 73.7067 E) in the month of November 2022 (Figure 1). The fish specimen was identified as per the taxonomic keys provided by Talwar and Jhingran (1991), Jayaram (2010), and Lal et al. (2015). The present study was approved by the Institutional Level Animal Ethics Committee of the College of Fisheries (Bihar Animal Sciences University), Kishanganj, India. The voucher specimen, along with tissue and DNA samples, is deposited in the repository of the Fisheries Resource Management Department of the College of Fisheries (Bihar Animal Sciences University), Kishanganj, Bihar, India, with accession number COF/1331/LR_01 (contact person: Rupam Samanta, rupamsamanta328@gmail.com). The total mitochondrial DNA was isolated from muscle tissue using the Alexgen DNA isolation kit as per the manufacturer’s instructions. The complete mitochondrial DNA sequencing was performed using Illumina’s high-throughput NovaSeq6000 with 2 × 150 bp paired-end chemistry. Details of the sequence depth and coverage map of the mitochondrial genome sequence generated in the present study are provided as supplementary material. A total of 11,913,402 reads generated (SRA accession no. SRR24210320) were *de novo* assembled into scaffolds using SPADes 3.15.4. A total of 1325 contigs were obtained, and the mitogenome was annotated with the help of MitoAnnotator (Zhu et al. 2023). MitoAnnotator allowed annotation of proteins, non-coding RNAs (tRNAs and rRNAs), and d-loop using blastx and blastn against known annotated mitochondrial proteins and rRNA genes, respectively. The maximum-likelihood (ML) phylogenetic tree was constructed based on the JTT matrix-based model (Jones et al. 1992) with the help of MEGA ver. 11.0.13 (Tamura et al. 2021), using a mitochondrial protein sequence dataset of all the 13 mitochondrial protein-coding genes (PCGs) of 21 closely related species and one outgroup species *Tor putitora* downloaded from the NCBI database (accession number AP011326). The initial tree(s) for the heuristic search were obtained automatically by applying the Neighbor-Joining and BioNJ algorithms to an estimated pair-wise distance matrix using the JTT model and then selecting the topology with a superior log likelihood value (–16516.24). Pairwise genetic divergence was also estimated using the Poisson correction model (Zuckerkandl and Pauling 1965). In this analysis, all ambiguous positions were removed for each sequence pair and a total of 3794 positions were used in the final dataset.

## Results

**Figure 2.**
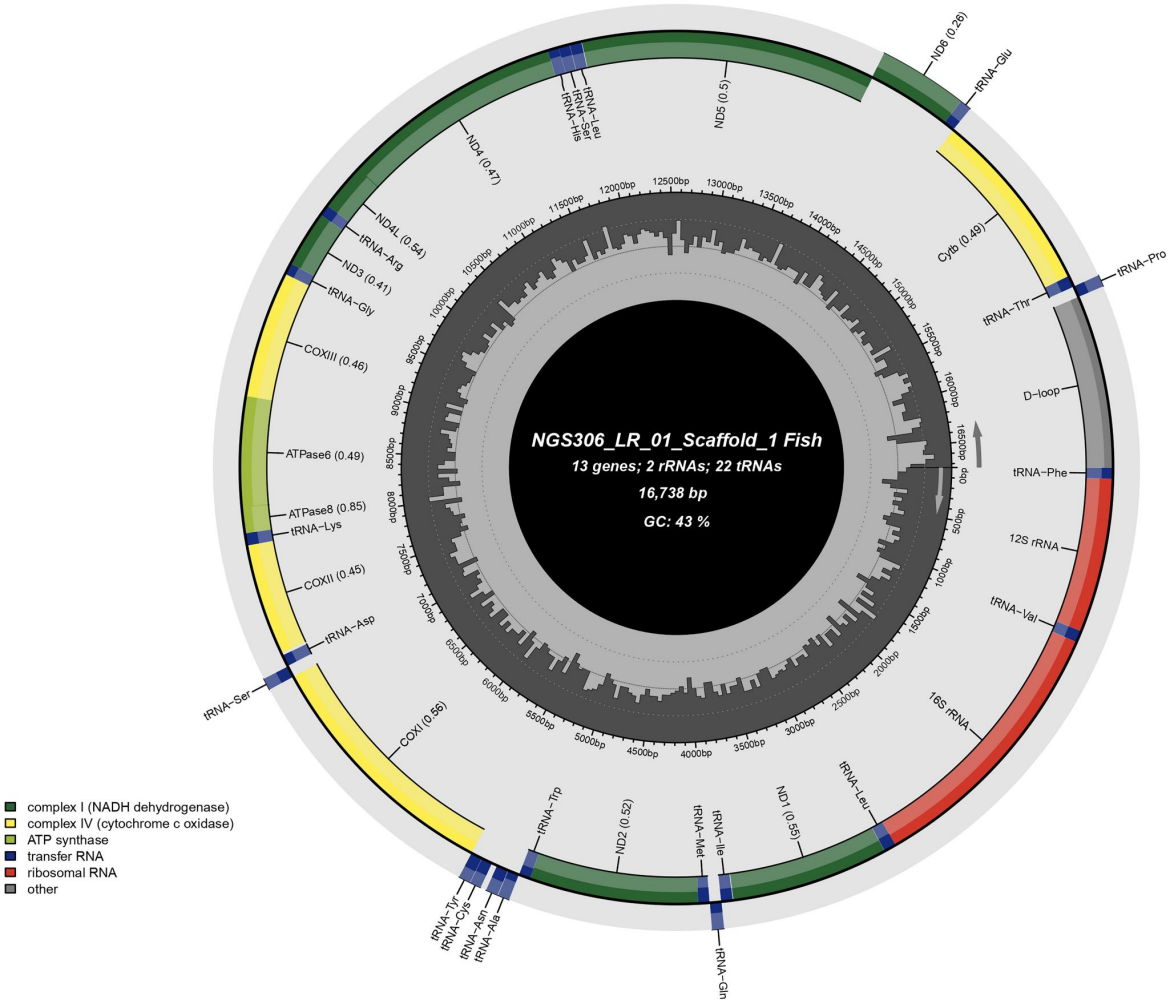
Map of *Labeo rajasthanicus* mitochondrial genome of 16,738 bp representing 13 protein-coding genes, 22 tRNA genes, two rRNA genes, and a 1069 bp long control region or D-loop.

**Figure 3.**
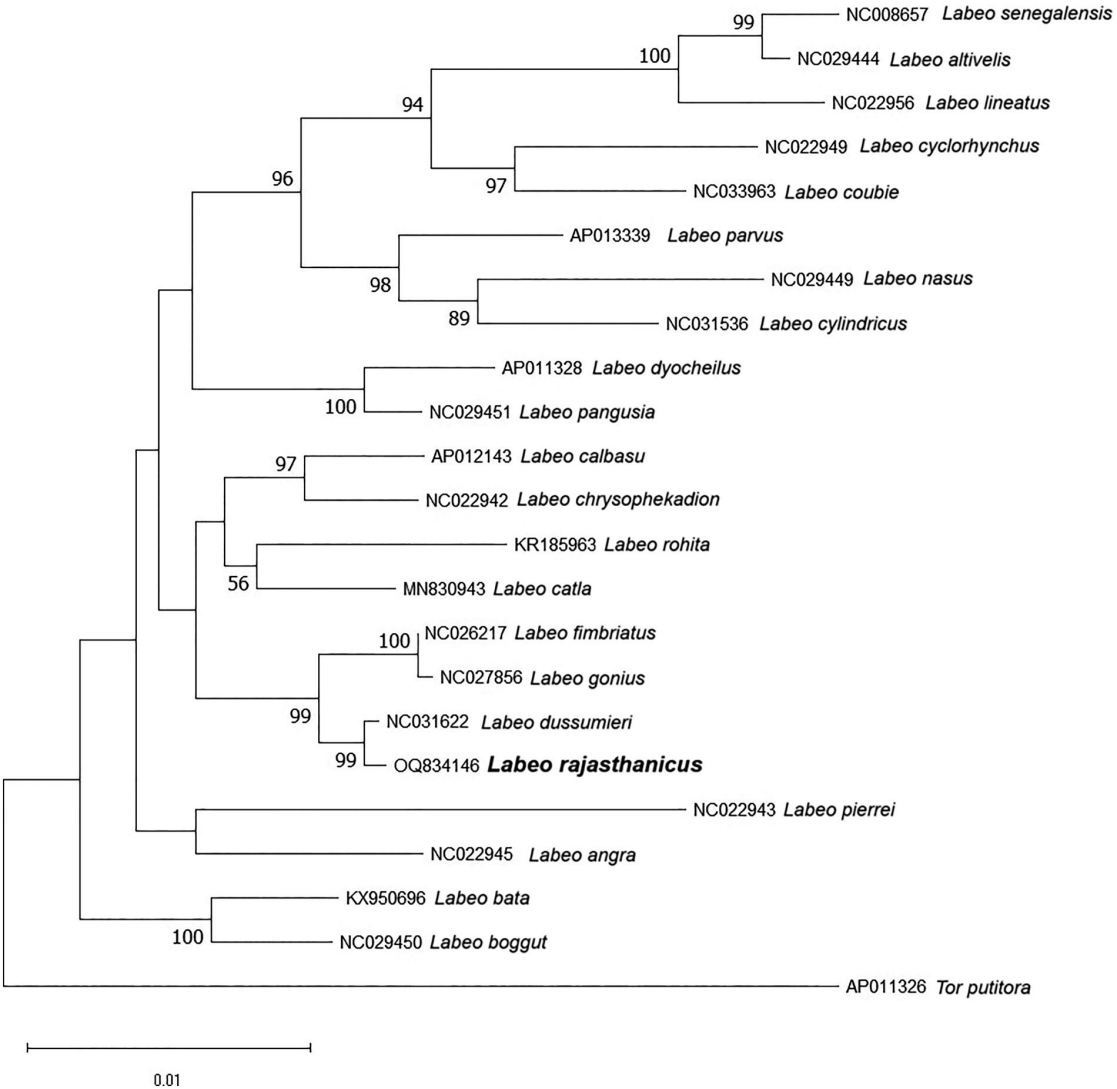
Maximum-likelihood phylogenetic tree using 13 mitochondrial protein-coding genes sequence of closely related 21 *Labeo* species and one out-group species *Tor putitora*. The percentage of trees in which the associated taxa clustered together is shown next to the branches and the tree is drawn to scale, with branch lengths measured in the number of substitutions per site. Sequences used in the present analysis are as follows: *Labeo rajasthanicus* (OQ834146), *Labeo senegalensis* (NC008657, Saitoh et al. 2006), *Labeo altivelis* (NC029444), *Labeo lineatus* (NC022956, Yang et al. 2012), *Labeo cyclorhynchus* (NC022949, Yang et al. 2012), *Labeo coubie* (NC033963), *Labeo parvus* (AP013339), *Labeo nasus* (NC029449), *Labeo cylindricus* (NC031536), *Labeo dyocheilus* (AP011328), *Labeo pangusia* (NC029451), *Labeo calbasu* (AP012143, Yang et al. 2012), *Labeo chrysophekadion* (NC022942, Yang et al. 2012), *Labeo rohita* (KR185963), *Labeo catla* (MN830943), *Labeo fimbriatus* (NC026217), *Labeo gonius* (NC027856), *Labeo dussumieri* (NC031622), *Labeo pierrei* (NC022943, Yang et al. 2012), *Labeo angra* (NC022945, Yang et al. 2012), *Labeo bata* (KX950696), *Labeo boggut* (NC029450), and *Tor putitora* (AP011326).

The complete mitogenome of *L. rajasthanicus* is 16,738 bp in length (GenBank accession no. OQ834146, Bio Project accession no. PRJNA957111), comprising 13 PCGs, 22 tRNA genes, two rRNA genes, and a 1069 bp long control region, or D-loop (Figure 2). The majority of genes were found on the H strand, except *ND6*, tRNA^Glu^, tRNA^Pro^, tRNA^Gln^, tRNA^Ala^, tRNA^Asn^, tRNA^Cys^, tRNA^Tyr^, and tRNA^Ser^, which were encoded on the L strand. *ATP6*, *ND2*, and *ND5* were found most divergent among all the PCGs. The GC% of the mitogenome of the studied species was found to be 42.76. D-loop was found highly A + T rich with 62.96% AT content. Two simple sequence repeats (SSRs) were also identified in the studied mitogenome. The ML phylogenetic tree (Figure 3) showed a distinct node of *L. rajasthanicus* supported by a high bootstrap value (99) and *L. rajasthanicus* had the closest relationship with *L. dussumieri* followed by *L. fimbriatus* and *L. gonius* but a distant relation with *L. boggut.* The pairwise evolutionary divergence was found to be minimum with *L. dussumieri* (0.13%) and maximum with *L. lineatus* (2.59%) whereas divergence with phenotypically closer *L. boggut* was estimated as 1.60%.

## Discussion and conclusions

The taxonomic status of *L. rajasthanicus* was considered ambiguous as its species validity was debated by some researchers, and it was considered a synonym of *L. boggut* (Jayaram and Dhas 2000). Our previous report (Lal et al. 2015) on the revision of the gonius subgroup confirmed the validity of this species. The present phylogenetic analysis using all the PCGs supports the same finding as *L. rajasthanicus* is well separated from *L. boggut* with high bootstrap value (99). Pairwise genetic divergence between *L. rajasthanicus* and *L. boggut* was also found higher (1.6%) than other valid species like *L. fimbriatus* (0.58%) and *L. gonius* (0.63%), thus further validating that *L. boggut* and *L. rajasthanicus* are two different species. Although low genetic divergence was found with *L. fimbriatus* and *L. gonius*, it is interesting to note that the nodes separating these species from *L. rajasthanicus* are supported by high bootstrap value (100). The low genetic divergence found in the present study may be due to recent divergence from a common ancestor (Amaral et al. 2007; Pereira et al. 2023) thus making them suitable to study the evolutionary history of *Labeo* species. Except from confirming the species validity of *L. rajasthanicus*, the present study will also be useful as a baseline reference genome for the reconstruction and annotation of the mitogenome of other *Labeo* species.

## Supplementary Material

Supplemental Material

Supplemental Material

## Data Availability

The genome sequence data that support the findings of this study are openly available in GenBank of the NCBI database at https://www.ncbi.nlm.nih.gov under accession no. OQ834146. The associated BioProject, SRA, and Bio-Sample accession numbers are PRJNA957111, SRP433314, and SAMN34246727, respectively.
