# Supplementary material for: Complete mitochondrial genome of golden variant of freshwater fish Labeo rajasthanicus (Cypriniformes: Cyprinidae): endemic to India
Source: Mitochondrial DNA B Resour. 2023 Dec 12;8(12):1364–7. doi: 10.1080/23802359.2023.2290128 (PMC10776062; doi:10.1080/23802359.2023.2290128)
Supplement: Supplemental Material [file TMDN_A_2290128_SM5401.docx]

**Supplementary Material**

Sequence depth and coverage map of mitochondrial genome sequence of *Labeo rajasthanicus* generated in the present study


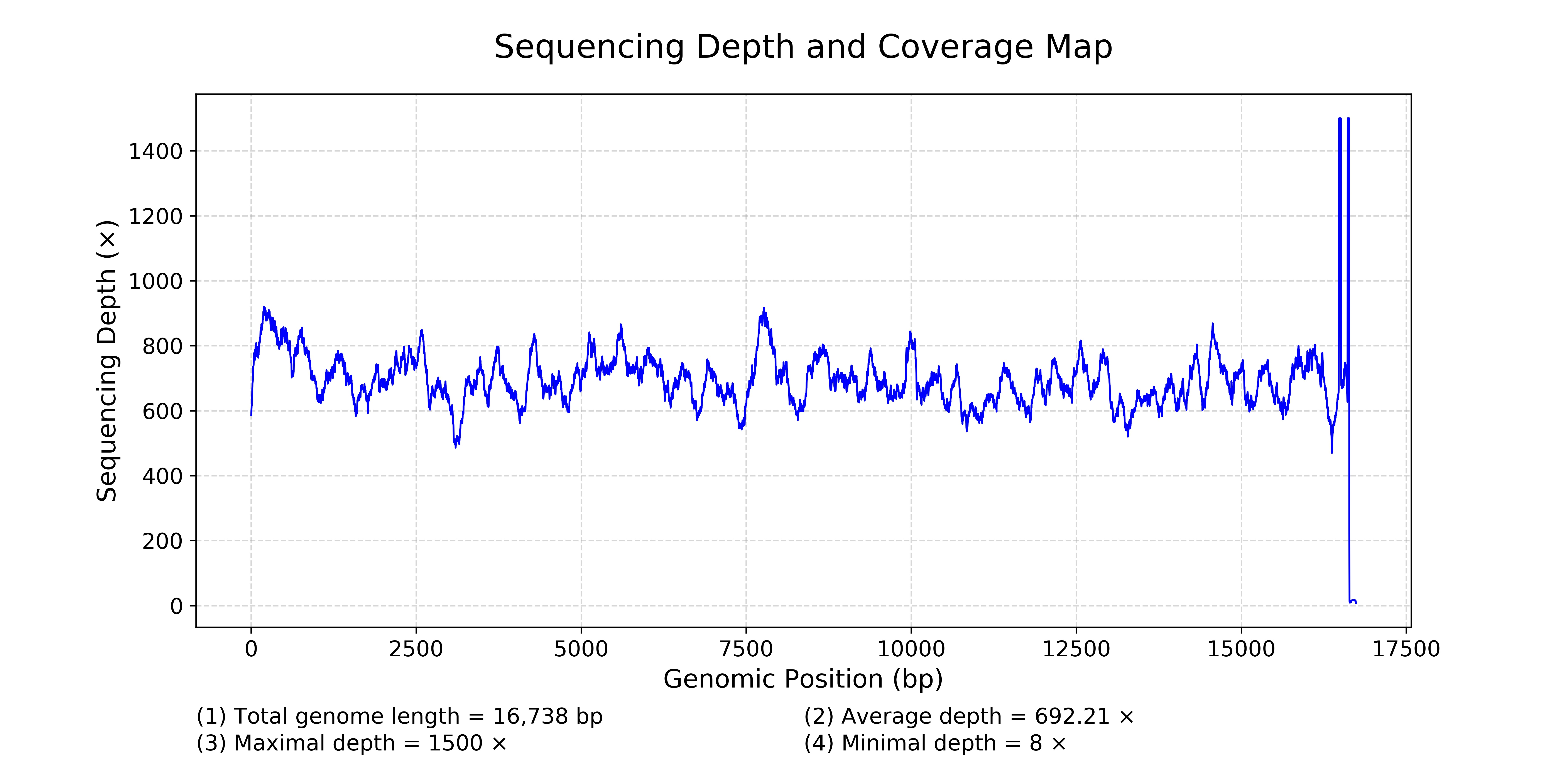


**Figure 1: Sequence depth and coverage map of Mitochondrial Genome Sequence of *Labeo rajasthanicus***
